# Impact of the Balloon Inflation Time and Pattern on the Coronary Stent Expansion

**DOI:** 10.1155/2019/6945372

**Published:** 2019-03-21

**Authors:** Jarosław Skowroński, Rafał Wolny, Jan Jastrzębski, Paweł Tyczyński, Karol Szlazak, Jerzy Pręgowski, Gary S. Mintz, Karolina Liżewska, Wojciech Świeszkowski, Zbigniew Chmielak, Adam Witkowski

**Affiliations:** ^1^Department of Interventional Cardiology and Angiology, Institute of Cardiology, Warsaw, Poland; ^2^Faculty of Materials Science and Engineering, Warsaw University of Technology, Warsaw, Poland; ^3^Cardiovascular Research Foundation, New York, USA

## Abstract

**Objectives:**

To assess the expansion pattern of coronary stents by using different balloon inflation times and pressures.

**Background:**

The selection of coronary stent size and its proper deployment is crucial in coronary artery interventions, having an impact on the success of the procedure and further therapy.

**Methods:**

Ten pairs of different stents were deployed under nominal pressure using sequential (5, 5, 10, and 10 seconds of repeated inflations, thus 30 seconds of summarized time) and continuous (30 seconds) deployment pattern. After each given time-point, intraluminal stent measurements were performed by optical coherence tomography (OCT) and intravascular ultrasound (IVUS).

**Results:**

Both in-stent diameters and cross-section areas (CSA) of paired stents measured by OCT at all sequential time-points were significantly smaller compared to given manufacturers charts' values (90% to 94% for diameters and 81% to 88% for CSA, p<0.05). Significant increase of in-stent diameter and CSA was observed across the step-by-step deployment pattern. In-stent lumen measurements were significantly larger when sequential deployment pattern was applied compared to continuous deployment. Additional measurements were also done for overlapping segments of stents, showing smaller in-stent measurements of the latter compared to nonoverlapping segments. Validation of OCT and IVUS measurements using a phantom metallic tube showed perfect reproducibility with OCT and overestimation with IVUS (8% for diameters and 16% for CSA).

**Conclusions:**

Stent diameter after deployment is time-dependent and not only pressure-dependent. Different stent expansion behavior, depending on the applied deployment pattern (sequential and nonsequential), was observed.

## 1. Introduction

Percutaneous coronary intervention (PCI) is a well-established method of revascularization. Introduction of coronary stents and their further developments resulted in the reduction of the risk of acute PCI complications (i.e., acute vessel closure, dissection) and improved long-term results through decrease of in-stent restenosis and stent thrombosis [1].

The optimal stent platform and the proper stent deployment are crucial for the satisfactory support of the treated coronary artery segment. Different stent shapes (tubular, coil, ring, mesh, and multidesign) have been developed to ensure radial strength required to resist external compressive forces, while maintaining flexible characteristics for crossing the vessel and lesion curvatures (trackability) [2].

Stent expansion is the result of a balance between compressive and expanding forces. The first is largely related to vessel characteristics: the wall stiffness and the plaque morphology. The latter depends on the properties of the expanding balloon (compliance), applied pressure, and deployment time. It is also influenced by stent design strut thickness and cell design. The impact of balloon characteristics and pressures has been extensively tested previously. Also, there is substantial evidence documenting that prolonged inflation (of at least 30 seconds) shows greater stent expansion than shorter inflation times [3–5]. Additionally, interesting mechanism of stent expansion has been shown, assuming that as long as inflation pressure decreases, stent expansion continues [6]. However the optimal protocol for the stent expansion time still needs to be established. According to in vivo intravascular imaging data a significant percentage of stents remain underexpanded despite a good angiographic appearance [7, 8]. Our bench study has two aims: (1) to compare the impact of two different balloon inflation time strategies on the stent expansion and (2) to evaluate the impact of high pressure balloon inflation in the overlapping stents region.

## 2. Materials and Methods

**Figure 1:**
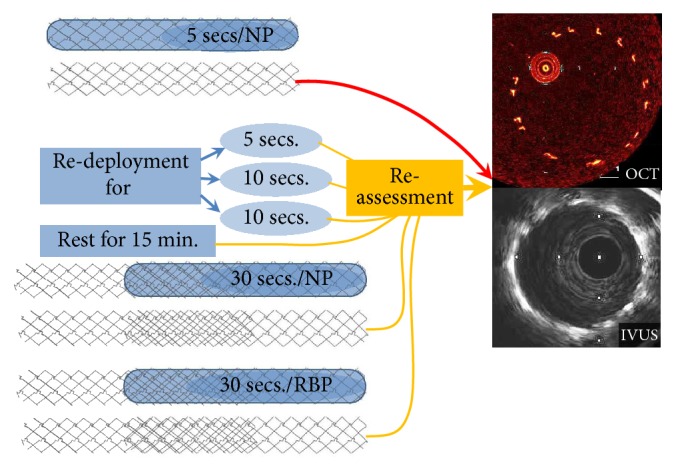
Scheme of study.* Abbreviations*: NP: nominal pressure, RBP: rated burst pressure, IVUS: intravascular ultrasonography, and OCT: optical coherence tomography.

Ten pairs of commercially available stents from six manufacturers, of identical type and diameter (different length, stent strut thickness, platform design and alloy, with or without antiproliferative drug coating between pairs) premounted on the balloons, were selected for the study (Table 1). Each stent was emerged in contrast medium (Iomeron 350, Bracco, Germany) without any additional support to avoid the potential influence of external forces on stent expansion. Stents were expended using ballon inflation device model SCW-BID1-20 (SCW Medicath LTD, Shenzhen, China) with a dial pointer. Each pair of stents was subsequently expanded under nominal pressure using two deployment algorithms. First paired stent was expanded with 4 short inflations lasting 5, 5, 10, and 10 seconds. Overall, the cumulative balloon expansion time in this multiple-time inflation (MTI) group was 30 seconds. Then, the second paired stent was partially placed into the first stent to create a double (overlapping region) and single layer of stent struts and then expanded again under nominal pressure directly for 30 seconds - one-time inflation group (OTI). Finally, a balloon positioned in the second paired stent (at overlapping and single segment) was deployed at rated burst pressure (RBP) for 30 seconds, as presented in the scheme (Figure 1).

One person (JS) was dedicated to careful balloon inflation at strictly predetermined pressure and time. If any drop in pressure was observed, it was simultaneously corrected by quick and precise reinflation to the nominal or RBP.

After each time-point, in-stent lumen image acquisitions by optical coherence tomography (OCT) and intravascular ultrasound (IVUS) were performed in the environment of contrast medium using Dragonfly C7 OCT imaging catheter (SJM, St Paul, MN, USA) and 40 MHz OptiCross Rotational IVUS imaging catheter (Boston Scientific, Marlborough, MA, USA), respectively.

Detailed* off-line* measurements of both in-stent diameter and cross-section area (CSA) from these two modalities were done by two experienced observers, blinded to the stent type and inflation parameters. For the second paired stent the measurements were done at the overlapping segment and at nonoverlapping segment, corresponding to the diameter and CSA after direct deployment time of 30 seconds. Then measurements were repeated after an additional 30 sec of inflation at RBP (Figure 1). All the results are presented as the ratio of actual diameter measurement and the expected diameter achievable at nominal pressure according to the compliance charts provided by the stent producer. The respective cross-sectional areas were derived from the actually measured and nominal diameters with use of *∏*r^2^ formula.

For each stent both OCT and IVUS cross-sections were analyzed at a proximal, mid, and distal segment. The final value was calculated as the mean of these three measurements derived from each cross-section.

Validation of OCT and IVUS measurements was performed using a phantom metallic tube with a defined inner diameter of 2.51mm and calculated CSA of 4.95mm^2^. Adequate image acquisition with both, the OCT and IVUS, was performed inside the lumen of the metallic phantom filled with same contrast medium.

Finally, three pairs of stents (Tables 3 and 4) were assessed by microcomputed tomography (SkyScan 1172, Bruker, Belgium). For the stent deployment the same as previous MTI and OTI protocols were applied, then the stents were scanned. The X-ray tube voltage was 40kV and current was 250*μ*A, and the resolution was 15*μ*m, performing a 180 degree rotation with a step size of 0.6 degrees and exposure time of 100 ms. CTAn (ver. 1.16.4.1+, Bruker, Belgium) and CTVol (ver. 2.3.2.0, Bruker, Belgium) software were used to process images and to obtain three-dimensional reconstructions. 800 consecutive cross-sections of each stent were analyzed.

Statistical analysis was performed using MedCalc software (Ostend, Belgium). Categorical variables were presented as percentages. Continuous variables were presented as means and standard deviation (SD). Continuous variables were compared using Student's t-test in case of normal distribution and Mann-Whitney test in case of nonnormal distribution. Statistical significance was assumed with p<0.05.

## 3. Results

The mean values of three serial measurements of inner lumen diameter and CSA of the metallic phantom tube were as follows: OCT 2.50mm and 4.93mm2; IVUS 2.56 and 5.37mm2, respectively. Taking into account the phantom's defined inner diameter of 2.51mm and calculated CSA of 4.95mm2, IVUS overestimated the lumen diameter by 2% and CSA by 8.5%.

**Figure 2:**
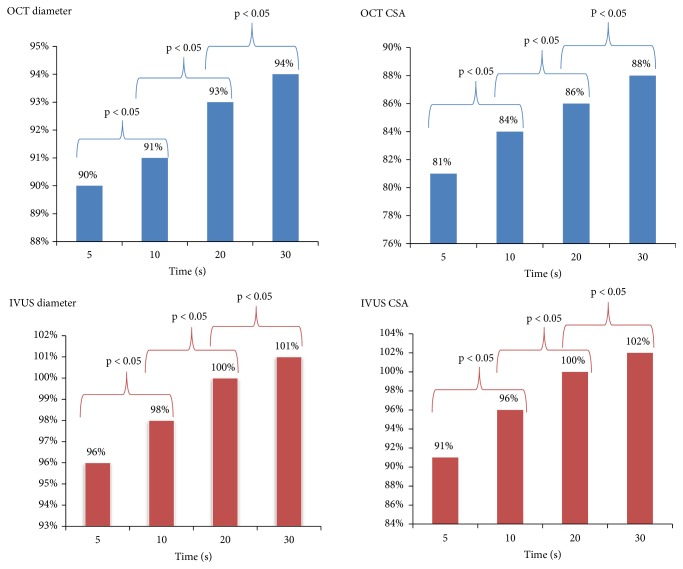
Comparison of in-stent diameter and CSA between 5 and 10, 10 and 20, and 30 and 30 seconds deployment time, respectively, by IVUS and OCT.* Abbreviations*: CSA: cross-section area; IVUS: intravascular ultrasonography; OCT: optical coherence tomography.

**Figure 3:**
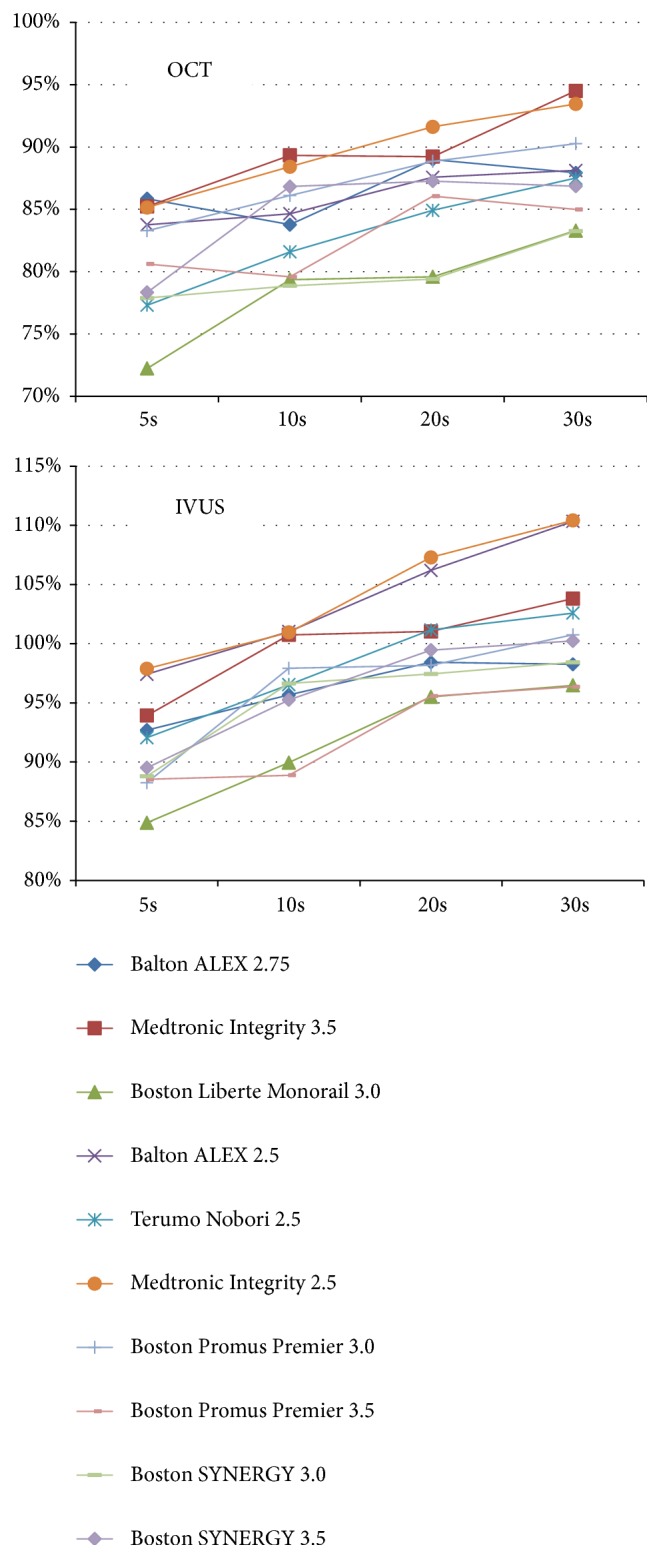
In-stent CSA after 5, 10, 20, and 30 seconds of deployment by OCT and IVUS.* Abbreviations*: CSA: cross-section area; IVUS: intravascular ultrasonography; OCT: optical coherence tomography.

**Figure 4:**
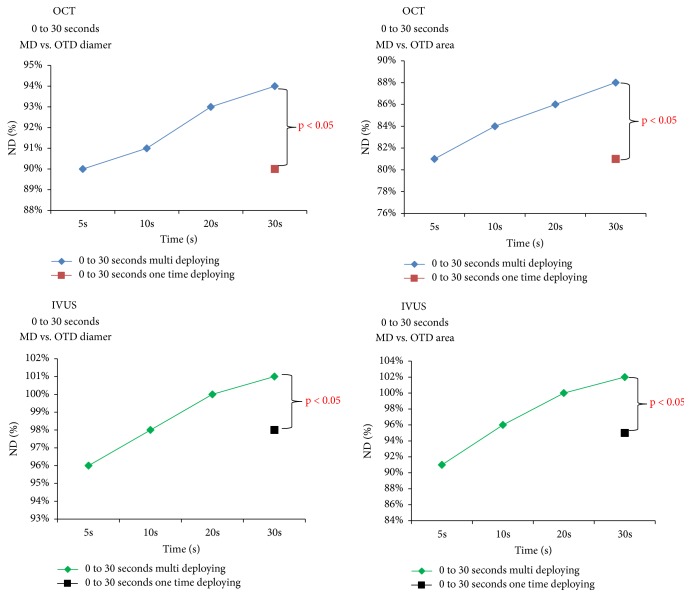
Comparison of in-stent diameter and CSA of multi-inflation time (MIT) 0 to 30 (5, 10, 20, 30 seconds of summarized time) to the one-time inflation (OTI) time 0 to 30 seconds by IVUS and OCT.* Abbreviations*: CSA: cross-section area; IVUS: intravascular ultrasonography; MD: multideploying; OCT: optical coherence tomography; OTD: one-time deploying.

**Figure 5:**
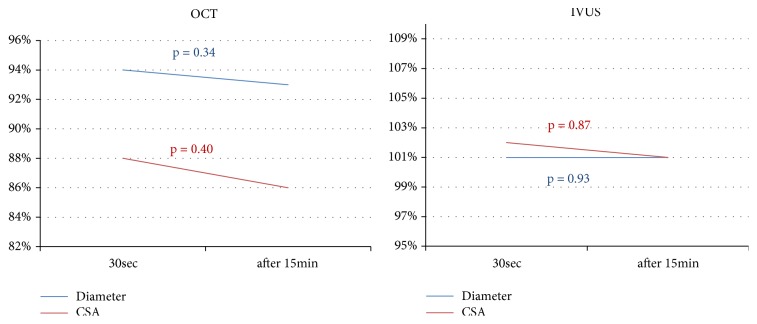
Comparison of in-stent diameter and CSA between 30 seconds deployment time and after 15 minutes of rest by IVUS and OCT.* Abbreviations*: CSA: cross-section area; IVUS: intravascular ultrasonography; OCT: optical coherence tomography.

The in-stent OCT and IVUS measurements obtained at respective time-points are summarized in Table 2 and in Figures 2 and 3. Measurements by OCT of either in-stent diameters and CSAs of the paired stents deployed under nominal pressures were significantly smaller at all sequential time-points as compared to the given compliance chart's values. The IVUS measurements were significantly closer to the chart's values (Table 2). Of note, a progressive increase of in-stent diameter and CSA were observed across subsequent steps (5-10-20-30 seconds) of the MTI protocol, regardless which technique of intravascular assessment (OCT or IVUS) was used (Figures 2 and 3). In-stent lumen measurements after 30 seconds of cumulative deployment time were larger for the MTI strategy. The final stent expansion indices for OTI strategy were similar to those obtained after the second step of MTI protocol. Interestingly, these observations were confirmed by both intravascular tools (Figure 4). There were no significant differences between in-stent measurements taken directly after 30 seconds of balloon inflation and after 15 minutes of rest (Figure 5). For further data see the supplementary materials, Tables 1 and 2.

**Figure 6:**
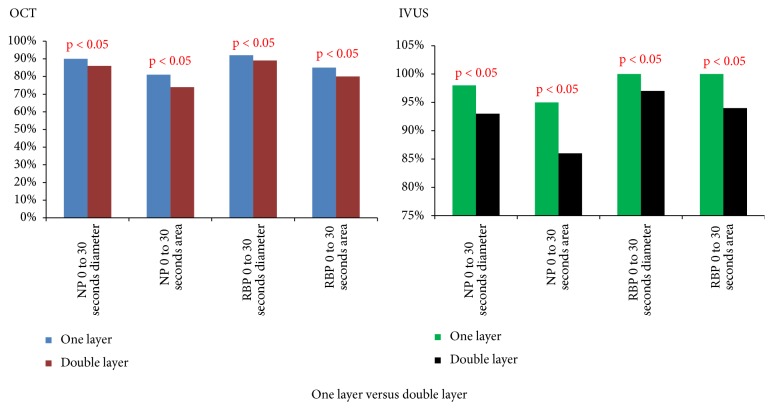
Comparison of in-stent diameter and CSA of the 0 to 30 second one-step deploying time in a one-layer stent to the diameter and CSA in double layer segments with 0 to 30 second one-step deploying time in the nominal pressure group and the RBP group by IVUS and OCT.* Abbreviations*: CSA: cross-section area; IVUS: intravascular ultrasonography; NP: nominal pressure; OCT: optical coherence tomography; RBP: rated burst pressure.

In case of overlapping stents' regions (stent-in-stent implantation), mean in-stent diameter and CSA at the level of a double layer of struts (overlapping segments) were significantly smaller compared to a single layer of struts (deployment with 30 seconds of balloon inflation), regardless of the pressure applied (nominal or RBP) in both OCT and IVUS (Table 2 and Figure 6). Direct comparison of corresponding OCT and IVUS measurements indicated larger latter values by 8% and 16% for all measured diameters and CSAs, (p<0.0001, both) respectively, which is in line with results derived from the metallic phantom model.

**Figure 7:**
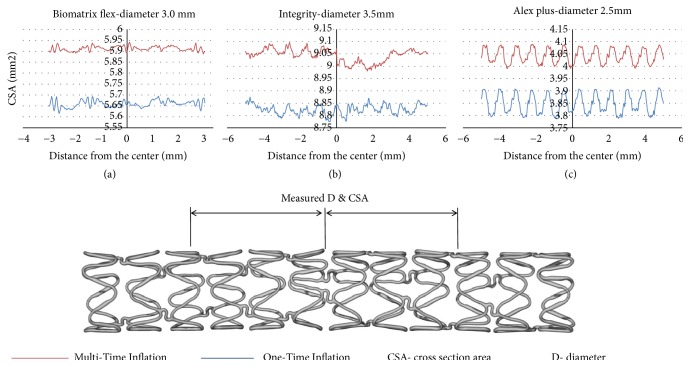
Microcomputed tomography CSA assessment of three stent deployed with MTI and OTI protocols. (a) stent Biomatrix flex 3.0 mm, (b) stent Medtronic Integrity 3.5 mm, (c) stent Balton Alex plus 2.5 mm.* Abbreviations*: CSA: cross-section area; D: diameter; IVUS: intravascular ultrasonography; MTI: multiple-time inflation; OCT: optical coherence tomography; OTI: one-time inflation.

Results from microcomputed tomography performed in 3 stent pairs are presented in Table 3 and Figure 7. Use of MTI deployment protocol resulted in increase of all mean lumen CSA (10%, 8%, and 3%) and mean diameter (4%, 4%, and 2%) of the stent, compared to OTI deployment protocol.

## 4. Discussion

The main finding of the current study is that multiple repetitive short balloon inflations result in a larger acute lumen gain than a single prolonged inflation of the same cumulative time.

It has been shown that stent underexpansion is a serious condition which may lead to potentially life-threatening stent thrombosis and increased risk of in-stent restenosis [9–11]. Therefore, optimal immediate PCI results are of crucial importance. It has been shown in previous in vivo studies that stent expansion is time-dependent and according to some authors even 60 seconds or more is required for an acceptable stent expansion* when clinically appropriate* [6, 12, 13]. On the other hand, the PCI operator may be reluctant to prolong the inflation time, especially in left main coronary artery, in hemodynamically unstable or critical coronary anatomy. Previous in vivo studies have shown that additional balloon inflations improve the stent expansion and apposition [14, 15]. Still, to the best of our knowledge the stent implantation protocol based on short subsequent balloon inflations compared to one prolonged inflation of the same time was not tested so far in bench study. We provided in vitro evidence suggesting that this protocol may result in a more favorable stent expansion than a single long inflation. The proposed strategy of several short inflations has the potential to be widely accepted and adopted by the interventional community as the ischemic intervals are short and unlikely to cause significant ischemia. An exception to this can be found in the report of Vallurupalli et al. which proved prolonged inflation time (average 103 seconds) is well tolerated in stable CAD patients [6]. One possible limitation of multiple inflation strategy is the risk of balloon dislocation that may cause injury to a nonstented vessel segment (i.e., geographic miss), especially in the presence of residual plaque burden, leading to edge restenosis [16, 17].

From the materials science point of view, the cause of bigger expansion of the stents subjected to sequential inflations than those subjected to one inflation is a phenomenon called ratcheting. It is defined as an accumulation of permanent strain in the material due to cyclic loading with nonzero mean stress. Initially, ratcheting occurs relatively quickly. Plastic deformation (permanent) accumulates in the material, causing the stents to expand more. As the number of cycles increases, however, ratcheting behavior gradually decreases [18]. This phenomenon leads to the dissipation of energy, which may result in the initiation and development of cracks.

A substantial number of PCIs require stent overlapping, especially in long and/or tandem coronary lesions. In cases of in-stent restenosis (ISR) a second stent implantation is an established treatment strategy. A double layer of stents may be associated with suboptimal deployment. In our study this issue was addressed by stent-in-stent deployment. In this case the mean in-stent diameter and CSA were significantly lower in comparison to single stent deployment. Even the prolonged, 30-second inflation time did not guarantee stent expansion to the nominal diameter indicated in the expansion chart. Such result was obtained in spite of optimal environment with absence of in-stent neointimal tissue, which additionally hampers stent expansion* in vivo*.

In our preliminary, ex vivo study two intravascular imaging methods were used to assess stent expansion. Of note, some measurement discrepancies between these two methods have been reported. In our study IVUS-derived measurements were systematically and significantly larger in comparison to OCT-derived measurements and dimensions obtained with OCT, as compared with IVUS, were noticeably more accurate in the assessment of the real size of the phantom. This is in line with previous data showing that IVUS overestimates the actual vessel dimensions. In the OPUS-CLASS Study both phantom measurements and in vivo results from multimodality intravascular assessments were reported [19]. In vivo the minimum lumen CSA measured by IVUS was significantly greater than that by OCT. In a phantom model, OCT-based MLA was equal to the actual lumen CSA of the phantom model, while IVUS overestimated the lumen CSA. In another study examining fixed human coronary arteries, both IVUS and OCT overestimated the lumen CSA compared with histomorphometry. In vivo the lumen dimensions obtained using IVUS were larger than those obtained using OCT [20]. Some advantage of OCT over IVUS in the assessment of stent length was also suggested. In the in vivo study by Liu et al. the difference between the actual stent lengths and the OCT-measured stent lengths was significantly smaller than that between the actual stent lengths and the IVUS-measured stent lengths [21]. In regard to the data mentioned above and the results of our study, especially by measurements performed using the phantom metallic model, it seems that OCT-derived measurements are closer to the real stent dimensions than IVUS-derived measurements and OCT rather than IVUS should be used as the reference methodology for in vivo stent expansion assessment. Therefore the observed difference between nominal stent diameter and the actual stent size after sequential balloon inflations is even more pronounced.

## 5. Limitations

This study has several limitations. First, this is a preliminary ex vivo study. Second, stents were tested only with use of nominal pressure inflation without the high pressure inflation used commonly in cath-labs in daily practice. Third, a relatively small number of stents with different diameters and lengths was studied; however, the 10 included pairs were representative for most commonly used materials and diameters. Fourth, the measurements were made in an ex vivo model which did not account for the external compressive forces of the vessel; however, even without external compressive forces the studied stents did not achieve their nominal diameter. Fifth, we used two commonly utilized measurement methods: IVUS and OCT, which are not as precise as laser measurements. Sixth, despite that only a single person was dedicated to careful and proper balloon inflation it is impossible to exclude transiently higher than expected pressure in the balloon. Moreover, the results cannot be fully clinically applicable due to the lack of fixed human coronary/ex vivo arterial model. We find that our results warrant a larger investigation with the use of precise measuring devices and a model accounting for external forces.

## 6. Conclusions

According to our bench study the short repetitive balloon inflations allow better stent expansion than single prolonged inflation of the same cumulative time. In vivo studies are needed to confirm this experimental finding.

## Figures and Tables

**Table 1:** Stent types used in the intravascular ultrasound and optical coherence tomography study.

Stent pairs	Stent name (manufacturer)	Platform material	Strut thickness, *μ*m	Type of coating drug	Nominal diameter, mm (pressure, atm.)	Diameter at RBP, mm (RBP, atm)	Length of paired stents, mm
1	Nobori (Terumo, Tokyo, Japan)	316L stainless steel	135	Biolimus	2.5 (8)	2.72 (16)	8
							28

2	Alex (Balton, Warsaw, Poland)	cobalt-chromium alloy	70	Sirolimus	2.5 (8)	2.81 (16)	8
							29
3					2.75 (8)	3.1 (16)	22
							22

4	Integrity (Medtronic, Inc., MN. USA)	cobalt-chromium alloy	91	-	2.55 (9)	2.8 (16)	14
							14
5					3.45 (9)	3.75 (16)	12
							12

6	Liberte Monorail (Boston Scientific, MA, USA)	316L stainless steel	97	-	3.03 (9)	3.48 (18)	20
							20

7	Promus Premier (Boston Scientific, MA, USA)	platinum-chromium alloy	81	Everolimus	2.95 (11)	3.17 (16)	38
							38
8					3.51 (11)	3.77 (16)	32
							32

9	Synergy (Boston Scientific, MA, USA)	platinum-chromium alloy	79	Everolimus	3.08 (11)	3.27 (16)	38
							38
10					3.55 (11)	3.79 (16)	32
							32

*Abbreviation:* RBP: rated burst pressure.

**Table 2:** Comparison of mean in-stent lumen diameters and cross-sectional areas at sequential deployment times and under different pressures (nominal and rated burst) measured with IVUS and OCT with nominal values (as per manufacturer's charts).

	In-stent diameter and CSA compared to nominal values (p value)			
Deployment time. sec.	OCT		IVUS	
	Diameter	CSA	Diameter	CSA
*Multitime inflation*				

5	90% (p<0.05)	81% (p<0.05)	96% (p<0.05)	91% (p<0.05)
10 (5, 5)	91% (p<0.05)	84% (p<0.05)	98% (p=0.11)	96% (p=0.11)
20 (5, 5, 10)	93% (p<0.05)	86% (p<0.05)	100% (p=0.42)	100% (p=0.42)
30 (5, 5, 10, 10)	94% (p<0.05)	88% (p<0.05)	101% (p=0.68)	102% (p=0.42)
Measurement after 15 min.	93% (p<0.05)	86% (p<0.05)	101% (p=1.00)	101% (p=1.00)

*One-time inflation*				

0->30	90% (p<0.05)	81% (p<0.05)	98% (p<0.05)	95% (p<0.05)
30 -> 60	92% (p<0.05)	85% (p<0.05)	101% (p=1.00)	102% (p=1.00)
DL 30	86% (p<0.05)	74% (p<0.05)	93% (p<0.05)	86% (p<0.05)
RBP 30	92% (p<0.05)	85% (p<0.05)	100% (p=0.42)	100% (p=0.42)
RBP DL 30	89% (p<0.05)	80% (p<0.05)	97% (p<0.05)	94% (p<0.05)

*Abbreviations:* CSA: cross-sectional area, DL: double layer; IVUS: intravascular ultrasound; OCT: optical coherence tomography; RBP: rated burst pressure.

**Table 3:** Lumen CSA and volume of three stents deployed with two protocols (MTI and OTI), as assessed by microcomputed tomography.

		STENT TYPES					
		Alex plus		Biomatrix flex		Integrity	
		CSA, mm2	D, mm	CSA, mm2	D, mm	CSA, mm2	D, mm
PROXIMAL	OTI	3.92	2.24	5.63	2.68	8.84	3.35
	MTI	4.38	2.36	5.88	2.74	9.06	3.40
	Diff.%	12%	6%	4%	2%	2%	1%

MID	OTI	3.84	2.21	5.06	2.54	8.39	3.27
	MTI	4.27	2.32	5.35	2.61	8.80	3.35
	Diff.%	11%	5%	6%	3%	5%	2%

DISTAL	OTI	3.93	2.24	5.38	2.62	8.85	3.36
	MTI	4.15	2.30	6.06	2.78	9.06	3.40
	Diff.%	6%	3%	13%	6%	2%	1%

MEAN	OTI	3.90	2.23	5.36	2.61	8.69	3.33
	MTI	4.27	2.33	5.76	2.71	8.97	3.38
	Diff.%	10%	4%	8%	4%	3%	2%

*Abbreviations:* CSA: cross section area; D: diameter; Diff.: difference; MTI: multitime inflation; OTI: one-time inflation.

**Table 4:** Stent types used in the microcomputed tomography study.

Stent pairs	Stent name (manufacturer)	Platform material	Strut thickness, *μ*m	Type of coating drug	Nominal diameter, mm (pressure, atm.)	Diameter at RBP, mm (RBP, atm)	Length of paired stents, mm
1	Biomatrix flex (Biosensoral Europe SA, Morges, Switzerland)	316L stainless steel	120	Biolimus	3.0 (6)	3.3(16)	11
							8

2	Integrity (Medtronic, Inc., MN. USA)	cobalt-chromium alloy	91	-	3.45 (9)	3.75 (16)	18
							15

3	Alex plus (Balton, Warsaw, Poland)	cobalt-chromium alloy	70	Sirolimus	2.5 (8)	2.81 (16)	25
							25

*Abbreviation:* RBP: rated burst pressure.

## Data Availability

The data used to support the findings of this study are available from the corresponding author upon request.

## Supplementary Materials

Supplementary Materials Table 1: comparison of in-stent average diameter and cross-sectional areas at sequential deployment times measured with IVUS and OCT to the first 5- second inflation. Table 2: comparison of in-stent average diameter and cross-sectional areas at sequential deployment times measured with IVUS and OCT between next inflations.
